# Changes in the immune system in experimental acanthamoebiasis in immunocompetent and immunosuppressed hosts

**DOI:** 10.1186/s13071-018-3108-x

**Published:** 2018-09-20

**Authors:** Natalia Łanocha-Arendarczyk, Agnieszka Kolasa-Wołosiuk, Iwona Wojciechowska-Koszko, Karolina Kot, Paulina Roszkowska, Barbara Krasnodębska-Szponder, Edyta Paczkowska, Bogusław Machaliński, Karolina Łuczkowska, Barbara Wiszniewska, Danuta Kosik-Bogacka

**Affiliations:** 1 Department of Biology and Medical Parasitology, Pomeranian Medical University in Szczecin, 70-204 Szczecin, Poland; 2 Department of Histology and Embryology, Pomeranian Medical University in Szczecin, 70-204 Szczecin, Poland; 3 Department of Microbiology, Immunology and Laboratory Medicine, Pomeranian Medical University in Szczecin, 70-204 Szczecin, Poland; 4 Department of General Pathology, Pomeranian Medical University in Szczecin, 70-204 Szczecin, Poland

**Keywords:** *Acanthamoeba* spp., Immunological status, Immunophenotype

## Abstract

**Background:**

Acanthamoebiasis is most often found in patients with immune deficiency, with infections facilitated by the intake of immunosuppressive drugs. The host immune response to *Acanthamoeba* spp*.* infection is poorly understood. Thus, in this study, we aimed to examine the course of *Acanthamoeba* spp*.* infection taking into account the host’s immunological status, including assessment of the hematological parameters, cytokine analysis, immunophenotypic changes in spleen populations, and histological spleen changes, which could help clarify some aspects of the immune response to acanthamoebiasis. In our experimental study, we used *Acanthamoeba* strain AM 22 isolated from the bronchoaspirate of a patient with acute myeloid leukaemia (AML) and atypical pneumonia symptoms.

**Results:**

*Acanthamoeba* spp*.* affected the hematological parameters in immunocompetent and immunosuppressed mice and induced a change in spleen weight during infection. Moreover, analysis of anti-inflammatory (IL-4 and IL-10) and pro-inflammatory (IL-17A and IFN-γ) cytokines produced by splenocytes stimulated with concanavalin A demonstrated that *Acanthamoeba* spp*.* induced a selective Th1, Th2 and Th17 response at later stages of the infection in immunocompetent hosts. In the case of hosts with low immunity, *Acanthamoeba* elicited robust Th1 cell-mediated immunity without the participation of Th17. We observed suppression of CD8+ and CD4+ T lymphocytes and CD3+CD4-CD8- double-negative (DN) T lymphocyte populations in the beginning, and in the case of CD3+/CD4+/CD8+ double-positive (DP) T cells in the final phase of *Acanthamoeba* spp*.* infection in hosts with low immunity. Also, CD4+T lymphocytes and CD3+/CD4+ and CD3+/CD8+ lymphocyte counts during each stage of acanthamoebiasis were shown to be upregulated.

**Conclusions:**

We demonstrated that analysis of the immune response and pathogenesis mechanisms of clinical isolates of *Acanthamoeba* spp*.* in an animal model not only has purely cognitive significance but above all, may help in the development of effective methods of pharmacological therapy especially in patients with low immunity.

## Background

Acanthamoebiasis is an opportunistic invasion caused by protozoans of the genus *Acanthamoeba* and is much more frequently noted in patients with immune deficiency, such as immunosuppressed patients following solid organ and bone marrow transplants and patients with HIV/AIDS [1]. Also, at risk are individuals with lymphoproliferative or hematologic disorders, diabetes mellitus, pneumonitis, renal failure, hepatic diseases, and gamma-globulinemia [1–3].

In immunosuppressed hosts, amoeba infections change the host-parasite relationships from oligosymptomatic and/or asymptomatic forms to acute disseminated parasitic infections affecting many organs, ultimately leading to the death of the host [4]. The widespread presence of *Acanthamoeba* makes them a significant threat to human health. After penetrating the central nervous system, lungs, cornea or human skin, they can cause granulomatous encephalitis (GAE), pneumonia (*Acanthamoeba pneumonia*), *Acanthamoeba* keratitis (AK), and cutaneous acanthamoebiasis (CA) [5, 6]. Several hundred cases of amoebic brain infections have been reported worldwide, with the first identified human case of GAE occurring in an immunosuppressed patient with Hodgkin’s disease [7]. The host immune response to *Acanthamoeba* spp*.* infection is poorly understood, even though 50–90% of people are known to be seropositive [8]. Immunocompetent patients are more likely to have brain invasions and secondary cutaneous lesions, while patients with low immunity more frequently have isolated primary cutaneous skin infections [9]. Immunocompetent patients develop a granulomatous reaction, while in immunosuppressed individuals, granuloma formation is weak, especially in HIV patients [10, 11].

Experimental and *in vitro* studies suggest the induction of innate and adaptive immune responses by *Acanthamoeba* spp., although there is a high discrepancy in the data [1, 12]. Brain *Acanthamoeba* spp*.* infection is a result of the host immune response and is most probably composed of CD4+ and CD8+ T cells, B lymphocytes, plasma cells and macrophages [2]. Deficiency in cell-mediated immunity is an important risk factor for GAE [13]. The factors that limit the activity of this protozoan parasite include neutrophils, macrophages and microglial cells. Moreover, their anti-parasitic effects are mediated in part by respiratory burst and nitric oxide under the influence of pro-inflammatory cytokines including interleukin-1, TNF and/or IFN [1, 10, 14, 15]. An *in vitro* study by Cano et al. [12] showed that ocular *Acanthamoeba keratitis* induced the proinflammatory macrophage phenotype characterised by the production of IL-12 and IL-6. Little is known about the pathogenesis of *Acanthamoeba* pneumonia including the complexity of immunological mechanisms. We do know that these parasites cause inflammation, e.g. in the lungs, with the coexistence of primary lesions in other organs and systems. Here, we focused on the immune system in both immunocompetent and in immunosuppressed hosts as a suitable animal model to study acanthamoebiasis in humans. This is the first experimental model induced by *Acanthamoeba* spp*.* isolated from the bronchoaspirate of a patient with acute myeloid leukaemia with atypical pneumonia symptoms [5]. Previously, we observed that this strain (AM 22) was pneumophilic, which may affect the expression and activity of cyclooxygenase-1 (COX-1) and cyclooxygenase-2 (COX-2), resulting in altered levels of their main products, prostaglandins (PGE_2_) and thromboxane B_2_ (TXB_2_), in the lungs of immunocompetent or immunosuppressed hosts [16]. Thus, in this study, we aimed to examine the course of *Acanthamoeba* spp*.* infection taking into account the host’s immunological status including assessment of the hematological parameters , cytokine analysis, immunophenotypic changes in spleen populations and histological spleen changes which could help clarify some aspects of the immune response to acanthamoebiasis.

## Methods

### Strain

The AM 22 strain isolated from bronchoaspirate of a 53-year-old male with acute septic shock was used in this study. The patient was in the blast crisis of acute myeloid leukemia and additionally was diagnosed with pneumonia. Based on analysis of a PCR-amplified 850 bp Ami fragment of 18S rRNA-gene the AM 22 strain was identified as *Acanthamoeba* genotype T16 [5]. Amoeba culture was grown on media with non-nutrient agar (NN Agar) coated with inactivated *Escherichia coli* bacteria, and incubated at 37 °C for 72 h. After the proliferation of *Acanthamoeba*, the trophozoites were washed with a sterile isotonic solution of 0.9% NaCl and prepared for intranasal inoculation of the test animals.

### Animal model

Our experimental model of acanthamoebiasis involved BALB/c mice (*n* = 96) aged about 6–10 weeks, divided into four groups: (i) Group C: immunocompetent animals not infected with *Acanthamoeba* spp*.*, (*n* = 18); (ii) Group A: immunocompetent animals infected with *Acanthamoeba* spp., (*n* = 30); (iii) Group CS: immunosuppressed animals not infected by *Acanthamoeba* spp.; immunosuppression induced by methylprednisolone (MPS) (*n* = 18); and (iv) Group AS: immunosuppressed animals infected by *Acanthamoeba* spp.; immunosuppression induced by MPS (*n* = 30).

For four days (-4, -3, -2, -1, 0) AS and CS animals were intraperitoneally administered 0.22 mg MPS (10 mg/kg) (Solu-Medrol, Pfizer, Puurs, Belgium, Europe MA EEIG), a steroid with immunosuppressive properties according to the methodology described by Markovitz et al. [17]. Such an algorithm made it possible to create an experimental model of tests under *in vivo* conditions, similar to patients with a reduced level of immunity. MPS is used in the treatment of acute organ rejection episodes. Animals from groups A and AS were given 3 μl of a suspension of *Acanthamoeba* trophozoites containing 10–20 thousand of the amoebae counted in Bürker chamber, intranasally. Mice from the C and CS groups received 3 μl of 0.9% NaCl intranasally. The animals were dissected at 8, 16 and 24 days after infection (dpi), depending on the onset of symptoms after intraperitoneal administration of sodium pentobarbital (2 ml/kg) (Euthasol vet, FATRO, Raamsdonksveer, The Netherlands). During the dissection, tissues and organs (including the spleen) were collected for immunological and histological analysis and adequately secured. The collected spleens were weighed.

### Laboratory blood test

Whole blood from the heart of the mice was sampled using the 1.2 ml blood collection system S Monovette (SARSTEDT AG&Co, Nümbrecht, Germany) containing K3EDTA as an anticoagulant. The study was carried out at the Clinical Central Laboratory in Szczecin. By means of a laser hematology analyzer, Sysmex XS-800i (Sysmex-Europe, Norderstedt, Germany), 11 parameters were measured in each sample: white blood cell count (WBC), number and percentage of lymphocytes (LYM), number and percentage of neutrophils (NEU), number and percentage of monocytes (MONO), number and percentage of eosinophils (EOS), number and percentage of basophils (BASO), red blood cell count (RBC), haemoglobin (HGB), hematocrit (HCT), and platelet count (PLT), as described by Kosik-Bogacka et al. [18].

### Flow cytometric immunophenotyping

**Fig. 1:**
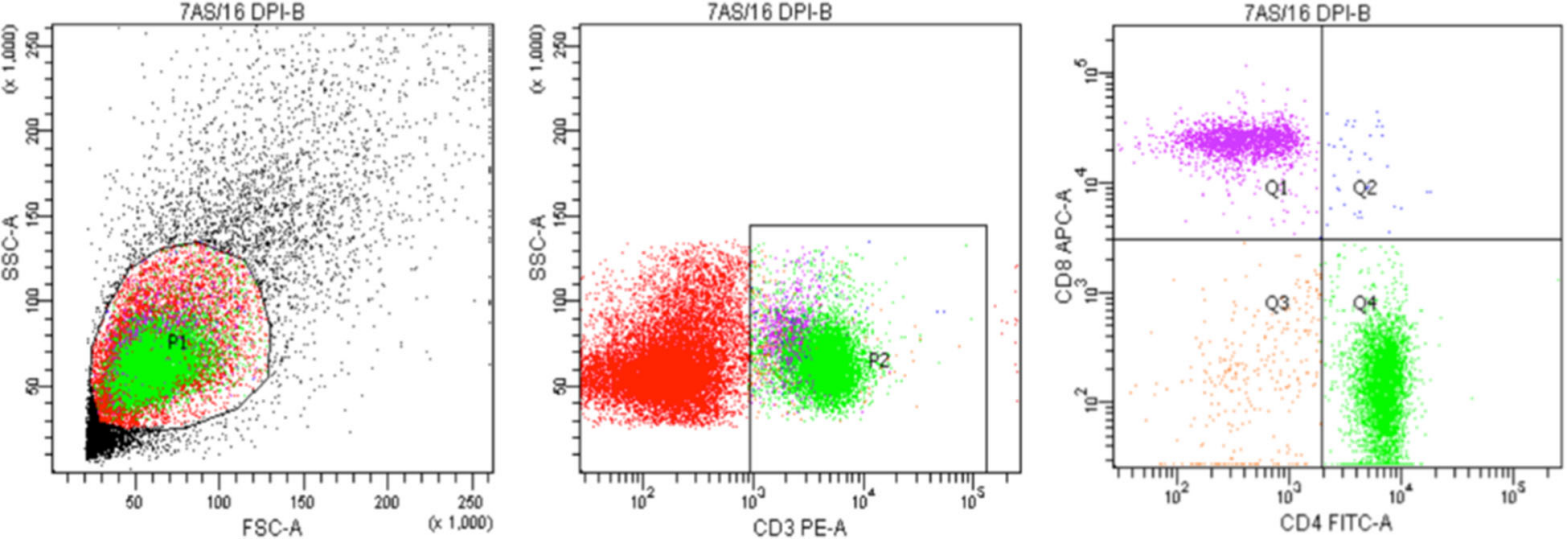
An example of a gating strategy used to identify sub-populations of T lymphocytes in the spleen using antigens CD3, CD4, CD8. Dot-plots show spleen cell gating on the side scatter (SSC) and forward scatter (FSC) (gate P1). Events contained by gate P2 were characterised by the presence on the surface of the CD3 antigen (CD3+ lymphocytes). Cells positive for the CD3 marker were further divided based on their expression of CD4 and CD8 antigen. Sub-populations of lymphocytes such as CD3+ (P2), CD3+/CD4+ (quadrant Q2+Q4), CD3+/CD8+ (Q1+Q2), CD3+/CD4+/CD8+ (Q2), CD3+/CD4-/CD8- (Q3) were analysed using this strategy

Immunophenotypic analysis of splenocytes by flow cytometry was undertaken as described by Kabat-Koperska et al. [19], and this test was only performed for immunosuppressed animals, including AS and CS groups. Spleens were collected during the section of the animals, then placed in Hank’s 1× balanced salt solutions, cut into pieces, pressed through nylon to sterile Roswell Park Memorial Institute medium (Sigma-Aldrich, St. Louis, USA ) containing phenol red and L-glutamine. The liquid was transferred to a centrifuge tube containing the same volume of RPMI 1640 medium, then centrifuged (2000× *g* for 20 min at room temperature). The middle cloud layer was placed in lymphocytes isolation medium and centrifuged (1000× *g* for 10 min at room temperature). The following monoclonal antibodies were used (mAb): PE Hamster Anti-Mouse CD3e (Clone 145-2C11), FITC Rat Anti-Mouse CD4 (Clone RM4-5), APC Rat Anti-Mouse CD8a (Clone 53-6.7) (BD Biosciences,San Jose, USA). Samples were subjected to acquisition using an LRS II flow cytometer (Becton Dickinsson, San Jose, CA, USA) and FacsDiva version 6.2 software (BD Biosciences). Appropriate isotype antibodies were used as controls (FITC Rat IgG2a κ Isotype Control, PE Hamster IgG1 κ Isotype Control, APC Rat IgG2a κ Isotype Control) (BD Biosciences). Typically, 30,000 events were acquired to determine the percentage of the examined sub-population within the spleen cell population. Analysis of sub-populations of lymphocytes in the spleen was based on the presence of CD3, CD4, and CD8 antigens on the cell surface (Fig. 1).

### Cytokine assays

Cell suspensions at a density of 2 × 10^6^ lymphocytes were placed in wells, 0.5 ml of cell suspension in 1 ml of incubation medium: RPMI, 10% bovine serum, 50 μg/ml of gentamycin and increasing amounts of concanavalin A (ConA, 0 μg/ml; 5 μg/ml) we estimated the functional response to this mitogen [19]. Incubation of lymphocytes in the well plates was carried out at 37 °C for 72 h under an atmosphere containing 5% CO_2_. After incubation, the cell suspensions were centrifuged at 1000× *g* for 10 min. The concentrations of cytokines (IL-4, IL-10, IL-17, INF-γ) were determined in the culture supernatants using enzyme-linked immunoassays according to the manufacturer’s recommendations (BD Biosciences, BD OptEIA Set Mouse IL-4, IL-10, INF-γ for IL-4, IL-10, INF-γ, respectively; IL-17A [homodimer] ELISA Ready-SET-GO for IL-17 (Affymetrix, Santa Clara, CA, USA). Results for each cytokine were expressed in μg/ml. Standard curves were derived from the cytokine standards supplied with the kit.

### Histology spleen examination

Spleen tissues were fixed in 10% formalin for at least 24 h and embedded in paraffin. Paraffin slides (3 μm) of the mice spleen were stained with hematoxylin-eosin, and a general histological examination was undertaken.

### Statistical analysis

The analysis used Statistica 10.0 software (StatSoft). The arithmetic means (AM), standard deviations of the AM (SD), and medians (Med) were calculated for each studied group. In order to determine compliance with the expected normal distribution of results, a Kolmogorov-Smirnov test with Lilliefors correction (*P* < 0.05) was used. Non-parametric tests (Kruskal-Wallis and Mann-Whitney U-test) were used as data deviated from a normal distribution. Differences between groups were analysed with the Mann-Whitney U-test for comparison of 2 groups and the Kruskal-Wallis test for comparison of 4 groups. The significance level was *P* < 0.05.

## Results

### Spleen weight

We observed a significantly lower total spleen weight in the *Acanthamoeba* spp. infected groups (immunocompetent and immunosuppressed) compared to control groups (C and CS) (Table 1). The weight of the spleen at 8 dpi was significantly lower in the group A than in the control (Mann-Whitney U-test, *U* = 30, *P* = 0.05), also at 16 dpi lower in the group A than in the group C (Mann-Whitney U-test, *U* = 41, *P* = 0.02) and lower in the group AS than in the group CS (Mann-Whitney U-test, *U* = 42.5, *P* = 0.02). At 24 dpi we observed significant differences between infected groups with different immunological status (A *vs* AS, Mann-Whitney U-test, *U* = 72, *P* = 0.02) (Table 1).

**Table 1:** Spleen weight (g) of male mice in control and *Acanthamoeba* spp*.* infected groups after 8, 16 and 24 days post-infection (dpi)

Parameter/group		Group A (*n* = 10)	Group C (*n* = 6)	Group AS (*n* = 10)	Group CS (*n* = 6)	Kruskal-Wallis test
Total spleen weight	AM ± SD	0.06 ± 0.03^a^	0.07 ± 0.008^a^	0.08 ± 0.06^b^	0.10 ± 0.01^b^	*H* = 29.81, *df* = 2, *P* = 0.001
	Median	0.07	0.07	0.1	0.1	
	Range	0.02–0.15	0.06–0.09	0.01–0.20	0.1–0.15	
8 dpi	AM ± SD	0.05 ± 0.02^c^	0.07 ± 0.01^c^	0.11 ± 0.04	0.10 ± 0.02	*H* = 22.78, *df* = 3, *P* = 0.001
	Median	0.06	0.07	0.10	0.10	
	Range	0.03– 0.07	0.06–0.08	0.1–0.2	0.1–0.15	
16 dpi	AM ± SD	0.05 ± 0.02^d^	0.07 ± 0.01^d^	0.04 ± 0.06^e^	0.10 ± 0.0^e^	*H* = 15.36, *df* = 3, *P* = 0.01
	Median	0.06	0.07	0.01	0.10	
	Range	0.02–0.07	0.06–0.08	0.01–0.20	0.10–0.10	
24 dpi	AM ± SD	0.08 ± 0.03^f^	0.08 ± 0.01	0.09 ± 0.03^f^	0.10 ± 0.0	*H* = 13.79, *df* = 3, *P* = 0.01
	Median	0.08	0.08	0.10	0.10	
	Range	0.05–0.15	0.07–0.09	0.01–0.10	0.10–0.10	

^a^*P* ≤ 0.03 for the significance of difference A *vs* C (Mann-Whitney U-test, *U* = 311),

^b^*P* ≤ 0.04 for the significance of difference AS *vs* CS (Mann-Whitney U-test, *U* = 297)

^c^*P* ≤ 0.05 for the significance of difference A *vs* C (Mann-Whitney U-test, *U* = 30)

^d^*P* ≤ 0.02 for the significance of difference A *vs* C (Mann-Whitney U-test, *U* = 41)

^e^*P* ≤ 0.02 for the significance of difference AS *vs* CS (Mann-Whitney U-test, *U* = 42.5)

^f^*P* ≤ 0.02 for the significance of difference A *vs* AS (Mann-Whitney U-test, *U* = 72)

*Abbreviations*: Group A, immunocompetent infected group; Group C, immunocompetent control group; Group AS immunosuppressed infected group; Group CS, immunosuppressed non-infected group; AM, arithmetic mean; SD, standard deviation

### Haematology results

We noted statistically significant differences in examined basic hematological parameters between control groups (C and CS) and *Acanthamoeba* spp*.*-infected immunocompetent mice (A) and infected mice with low immunity (AS) (Table 2). We observed higher lymphocyte count in the group AS than in the group CS (0.92 G/l *vs* 0.65 G/l, Mann-Whitney U-test, *U* = 78, *P* = 0.01). There were significantly lower monocyte counts in the group A than in the group AS (0.11 G/l *vs* 0.36 G/l, Mann-Whitney U-test, *U* = 51, *P* = 0.02) and higher in the group AS than in the group CS (0.36 G/l *vs* 0.06 G/l, Mann-Whitney U-test, *U* = 57.5, *P* = 0.03). The basophile count was significantly higher in the group AS, than in the group CS (0.23 G/l *vs* 0.05 G/l, Mann-Whitney U-test, *U* = 76, *P* = 0.01). We found higher platelet count in the group A than in the group C (633.3 G/l *vs* 481.4 G/l, Mann-Whitney U-test, *U* = 52, *P* = 0.05) (Table 2).

**Table 2:** Hematology results of *Acanthamoeba* spp*.* infected immunocompetent, immunosuppressed and control groups

Parameter/group		Group A (*n* = 10)	Group C (*n* = 10)	Group AS (*n* = 10)	Group CS (*n* = 10)	Kruskal-Wallis test
WBC (G/l)	AM ± SD	1.83 ± 0.55	1.38 ± 0.33	1.57 ± 0.85	1.92 ± 1.05	*H* = 2.03, *df* = 3, *P* = 0.57
	Median	1.89	1.27	1.53	1.89	
	Range	1.14–2.74	1.12–1.85	0.63–3.41	0.40–3.60	
LYM (G/l)	AM ± SD	1.11 ± 0.27	1.11 ± 0.25	0.92 ± 0.56^a^	0.65 ± 0.38^a^	*H* = 12.04, *df* = 3, *P* = 0.04
	Median	1.08	1.05	0.84	0.55	
	Range	0.78–1.51	0.89–1.47	0.00–1.68	0.32–1.08	
NEU (G/l)	AM ± SD	0.01 ± 0.01	0.02 ± 0.01	0.03 ± 0.07	0.01 ± 0.01	*H* = 3.32, *df* = 3, *P* = 0.47
	Median	0.01	0.01	0.01	0.01	
	Range	0.00–0.01	0.00–0.02	0.00–0.24	0.00–0.02	
MONO (G/l)	AM ± SD	0.11 ± 0.12^b^	0.08 ± 0.13	0.36 ± 0.87^b,c^	0.06 ± 0.10^c^	*H* = 6.48, *df* = 3, *P* = 0.02
	Median	0.04	0.02	0.05	0.05	
	Range	0.01–0.27	0.00–0.27	0.00–2.80	0.00–0.30	
EOS (G/l)	AM ± SD	0.02 ± 0.01		0.06 ± 0.19	0.17 ± 0.28	*H* = 0.71, *df* = 3, *P* = 0.86
	Median	0.01		0.03	0.01	
	Range	0.00–0.02	0	0.01–0.60	0.01–0.50	
BASO (G/l)	AM ± SD	0.27 ± 0.13	0.13 ± 0.07	0.23 ± 0.17^d^	0.05 ± 0.08^d^	*H* = 7.75, *df* = 3, *P* = 0.01
	Median	0.23	0.13	0.24	0.02	
	Range	0.14–0.49	0.09–0.49	0.01–0.61	0.01–0.20	
RBC (T/l)	AM ± SD	9.30 ± 0.37	9.64 ± 0.10	9.18 ± 0.42	8.47±3.19	*H* = 4.41, *df* = 3, *P* = 0.22
	Median	9.34	9.62	9.24	9.59	
	Range	8.7–9.7	9.5–9.8	8.3–9.68	9.02–10.1	
HGB (mmol/l)	AM ± SD	9.01 ± 0.34	9.37 ± 0.15	8.91 ± 0.33	9.16 ± 0.30	*H* = 4.92, *df* = 3, *P* = 0.17
	Median	9.0	9.04	9.0	9.27	
	Range	8.6–9.6	9.2–9.5	8.30–9.2	8.70–9.6	
HCT (L/l)	AM ± SD	0.41 ± 0.009	0.43 ± 0.01	0.41 ± 0.02	0.42 ± 0.01	*H* = 2.02, *df* = 3, *P* = 0.57
	Median	0.41	0.43	0.41	0.41	
	Range	0.40–0.43	0.41–0.43	0.38–0.43	0.40–0.43	
PLT (G/l)	AM ± SD	633.3 ± 176.6^e^	481.4 ± 277.6^e^	866.8 ± 60.3	912.0 ± 109.3	*H* = 4.60, *df* = 3, *P* = 0.02
	Median	646.5	355.0	889.5	880.0	
	Range	432.0–932.0	204.0–808.0	746.0–950.0	803.0–1120.0	

^a^*P* ≤ 0.01 for the significance of difference AS *vs* CS (Mann-Whitney U-test, *U* = 78)

^b^*P* ≤ 0.02 for the significance of difference A *vs* AS (Mann-Whitney U-test, *U* = 51)

^c^*P* ≤ 0.03 for the significance of difference AS *vs* CS (Mann-Whitney U-test, *U* = 57.5)

^d^*P* ≤ 0.01 for the significance of difference AS *vs* CS (Mann-Whitney U-test, *U* = 76)

^e^*P* ≤ 0.05 for the significance of difference A *vs* C (Mann-Whitney U-test, *U* = 52)

*Abbreviations*: Group A, immunocompetent infected group; Group C, immunocompetent control group; Group AS immunosuppressed infected group; Group CS, immunosuppressed non-infected group; G, giga; AM, arithmetic mean; SD, standard deviation

### Cytokine assays

Analysis of cytokine profile after incubation of the lymphocytes from the spleen with increasing doses of concanavalin A (ConA) (0 and 5 μg/ml) found higher level of IL-4 (anti-inflammatory cytokine) after administration of a higher dose of ConA (5 μg/ml) at 16 dpi in the group A compared to AS (Mann-Whitney U-test, *U* = 22.5, *P* = 0.02). We observed an increased level of IL-10 (anti-inflammatory cytokine) with a higher dose of Con A in both infected groups (A and AS) at 16 dpi (Mann-Whitney U-test; group A *vs* C: *U* = 22, *P* = 0.05; AS *vs* CS: *U* = 25, *P* = 0.01; A *vs* AS: *U* = 16, *P* = 0.01). There were significantly higher levels of IL-17A (proinflammatory cytokine) with a higher dose of Con A at 16 dpi in group A compared to group C (Mann-Whitney U-test, *U* = 22.5, *P* = 0.02) and with a lower dose of ConA at 24 dpi in group A *vs* C (Mann-Whitney U-test, *U* = 13.5, *P* = 0.05). We noted decreased level of IFN-γ (proinflammatory cytokine) with a higher dose of ConA in infected immunocompetent group (A) compared to infected immunosuppressed group (AS) at 24 dpi (Mann-Whitney U-test, *U* = 11, *P* = 0.05) (Table 3).

**Table 3:** Cytokine profile after incubation of lymphocytes from the spleen with different doses of concanavalin A, ConA (0 μg/ml; 5 μg/ml)

Parameter/group		Group A (*n* = 10)	Group C (*n* = 6)	Group AS (*n* = 10)	Group CS (*n* = 6)	Kruskal-Wallis test
IL-4						
8 dpi	ConA0	0	0	0	0	*P* = 1.0
	ConA5	0.52 ± 4.33	0.26 ± 0.59	0	0	*H* = 2.16, *df* = 3, *P* = 0.54
16 dpi	ConA0	0	0	0	1.94 ± 4.33	*H* = 3.18, *df* = 3, *P* = 0.39
	ConA5	50.35 ± 51.75^a^	0	0^a^	0	*H* = 11.22, *df* = 3, *P* = 0.002
24 dpi	ConA0	0	0	1.28 ± 2.86	0	*H* = 3.00, *df* = 3, *P* = 0.31
	ConA5	1.19 ± 2.67	0.66 ± 1.47	0	0	*H* = 21.00, *df* = 3, *P* = 0.54
IL-10						
8 dpi	ConA0	84.63 ± 46.72	149.98 ± 128.88	83.06 ± 37.32	58.90 ± 26.14	*H* = 3.94, *df* = 3, *P* = 0.27
	ConA5	168.43 ± 180.88	212.92 ± 180.20	139.47 ± 125.42	86.16 ± 12.56	*H* = 4.07, *df =* 3, *P* = 0.25
16 dpi	ConA0	128.27 ± 65.83	78.98 ± 58.82	78.60 ± 46.47	69.07 ± 15.19	*H* = 4.62, *df* = 3, *P* = 0.34
	ConA5	511.09 ± 40.41^b, c^	67.42 ± 16.98^b^	276.18 ± 224.72^c,d^	81.01 ± 19.34^d^	*H* = 10.30, *df* = 3, *P* = 0.01
24 dpi	ConA0	14.94 ± 33.40	19.99 ± 99.96	0	0	*H* = 2.16, *df* = 3, *P* = 0.55
	ConA5	82.13 ± 82.73	36.54 ± 74.12	0	230.67 ± 515.76	*H* = 3.73, *df* = 3, *P* = 0.29
IL-17 A						
8 dpi	ConA0	0	0	0	0	*P* = 1.0
	ConA5	13.86 ± 30.98	0	0	0	*H* = 3.00, *df* = 3, *P* = 0.39
16 dpi	ConA0	0	0	0	0.75 ± 1.68	*H* = 4.00, *df* = 3, *P* = 0.56
	ConA5	78.60 ± 55.28^e^	0^e^	0	5.24 ± 7.29	*H* = 11.22, *df* = 3, *P* = 0.01
24 dpi	ConA0	9.97 ± 13.97^f^	2.05 ± 1.54^f^	0	0	*H* = 8.44, *df* = 3, *P* = 0.04
	ConA5	13.27 ± 16.80	1.81 ± 2.06	32.85 ± 37.08	38.77 ± 52.46	*H* = 2.60, *df* = 3, *P* = 0.45
IFN-γ						
8 dpi	ConA0	0	0	0	0	*P* = 1
	ConA5	0	0	0	0	*P* = 1
16 dpi	ConA0	0	24.0 ± 53.66	0	0	*H* = 3.00, *df* = 3, *P* = 0.39
	ConA5	0	0	0	0	*P* = 1
24 dpi	ConA0	0	0	0	0	*P* = 1
	ConA5	594.21 ± 772.32^g^	0	939.29 ± 768.24^g^	546.31 ± 738.50	*H* = 8.80, *df* = 3, *P* = 0.03

^a^*P* ≤ 0.02 for the significance of difference A *vs* AS (Mann-Whitney U-test, *U* = 22.5)

^b^*P* ≤ 0.05 for the significance of difference A *vs* C (Mann-Whitney U-test, *U* = 22)

^c^*P* ≤ 0.01 for the significance of difference A *vs* AS (Mann-Whitney U-test, *U* = 16)

^d^*P* ≤ 0.01 for the significance of difference AS *vs* CS (Mann-Whitney U-test, *U* = 25)

^e^*P* ≤ 0.02 for the significance of difference A *vs* C (Mann-Whitney U-test, *U* = 22.5)

^f^*P* ≤ 0.05 for the significance of difference A *vs* C (Mann-Whitney U-test, *U* = 13.5)

^g^*P* ≤ 0.05 for the significance of difference A *vs* AS (Mann-Whitney U-test, *U* = 11)

*Abbreviations*: Group A, immunocompetent infected group; Group C, immunocompetent control group; Group AS immunosuppressed infected group; Group CS, immunosuppressed non-infected group; AM, arithmetic mean; SD, standard deviation

### Immunophenotyping results

Immunophenotyping analysis of splenocytes by flow cytometry was only performed for immunosuppressed animals including AS and CS groups. After splenocytes flow cytometry analysis we observed increased CD3+/CD4+ lymphocyte counts in the group AS at 24 dpi (Mann-Whitney U-test, *U* = 21, *P* = 0.05) and increased CD3+/CD8+ lymphocyte counts in the group AS at 16 dpi (Mann-Whitney U-test, *U* = 25, *P* = 0.01). We noted decreased populations of double negative cells (CD3+/CD4-/CD8-) at 8 dpi in the group AS (Mann-Whitney U-test, *U* = 24, *P* = 0.02) and increased populations of double positive cells (CD3+/CD4+/CD8+) at 24 dpi in the group AS (Mann-Whitney U-test, *U* = 19, *P* = 0.03) (Table 4). In addition, the percentage of CD3+ T cells, CD4+ T cells and CD8+ T cells, the CD4+ / CD8+ cell ratio and the CD4-/CD8- cell population ratio were determined. We observed decreased CD3+ lymphocyte counts in *Acanthamoeba* spp.-infected groups at 8 dpi (Mann-Whitney U-test, *U* = 23, *P* = 0.04), but increased at 24 dpi (Mann-Whitney U-test, *U* = 22, *P* = 0.05). We noted increased CD4+ (Mann-Whitney U-test, *U* = 25, *P* = 0.01) and decreased CD8+ lymphocyte counts (Mann-Whitney U-test, *U* = 25, *P* = 0.05) in the group AS at 8 dpi. There was significantly lower CD4-/CD8- lymphocyte ratio counts in the group AS compared with the group CS (Mann-Whitney U-test, *U* = 22, *P* = 0.05) at 8 dpi (Table 5).

**Table 4:** Immunophenotyping results in % (spleen) of *Acanthamoeba* spp.-infected immunosuppressed mice and control mice with low immunity

Parameter/group			Group AS (*n* = 10)	Group CS (*n* = 6)	Mann-Whitney U-test
CD3+/CD4+ (T lymphocytes) (%)	8 dpi	AM ± SD	22.67 ± 0.13	24.47 ± 0.09	*U* = 16, *P* = 0.53
		Median	22.87	25.57	
		Range	18.62–26.69	21.51–26.73	
	16 dpi	AM ± SD	28.87 ± 0.05	27.51 ± 0.20	*U* = 15, *P* = 0.09
		Median	25.09	25.78	
		Range	23.11–26.19	22.27–36.82	
	24 dpi	AM ± SD	25.17 ± 0.43	16.68 ± 0.18	*U* = 21, *P* = 0.05
		Median	22.17	17.35	
		Range	14.67–43.74	12.04–20.34	
CD3+/CD8+ (%)	8 dpi	AM ± SD	5.25 ± 0.14	6.16 ± 0.37	*U* = 14, *P* = 0.83
		Median	5.21	6.03	
		Range	4.44–6.28	4.23–9.89	
	16 dpi	AM ± SD	7.40 ± 0.05	4.55 ± 0.24	*U* = 25, *P* = 0.01
		Median	7.51	4.19	
		Range	6.83–7.94	3.39–6.36	
	24 dpi	AM ± SD	4.23 ± 0.60	4.10 ± 0.17	*U* = 19, *P* = 0.21
		Median	5.54	3.83	
		Range	0.05–7.45	3.35–5.16	
CD3+/CD4+/CD8+ (DP %)	8 dpi	AM ± SD	1.13 ± 0.19	1.82 ± 0.29	*U* = 21, *P* = 0.09
		Median	1.18	1.87	
		Range	0.83–1.35	0.96–2.36	
	16 dpi	AM ± SD	1.52 ± 0.24	1.61 ± 0.38	*U* = 13, *P* = 0.99
		Median	1.66	1.61	
		Range	0.98–1.94	1.17–2.6	
	24 dpi	AM ± SD	2.71 ± 0.89	1.32 ± 0.35	*U* = 19, *P* = 0.03
		Median	1.63	1.07	
		Range	1.1–7.47	0.96–2.03	
CD3+/CD4-/CD8- (DN %)	8 dpi	AM ± SD	0.12 ± 0.21	0.21 ± 0.18	*U* = 24, *P* = 0.02
		Median	0.12	0.23	
		Range	0.1–0.16	0.16–0.24	
	16 dpi	AM ± SD	0.14 ± 0.10	0.09 ± 0.65	*U* = 20, *P* = 0.14
		Median	0.14	0.09	
		Range	0.11–0.17	0.03–0.18	
	24 dpi	AM ± SD	0.16 ± 0.59	0.11 ± 0.26	*U* = 19, *P* = 0.15
		Median	0.22	0.11	
		Range	0.004–0.24	0.07–0.13	

*Abbreviations*: Group A, immunocompetent infected group; Group C, immunocompetent control group; Group AS immunosuppressed infected group; Group CS, immunosuppressed non-infected group; AM, arithmetic mean; SD, standard deviation DP, CD3+/CD4+/CD8+ double-positive T cells; DN, CD3+CD4−CD8− double-positive T cells

**Table 5:** The percentage of T lymphocyte sub-populations in the spleen of *Acanthamoeba* spp.-infected immunosuppressed mice and control mice with low immunity

Parameter/group			Group AS (*n* = 10)	Group CS (*n* = 6)	Mann-Whitney U-test
CD3+ (T lymphocytes)%	8 dpi	AM ± SD	29.21 ± 3.23	33.99 ± 2.60	*U* = 23, *P* = 0.04
		Median	29.78	34.63	
		Range	24.75–33.23	30.94–36.46	
	16 dpi	AM ± SD	32.77 ± 1.62	36.07 ± 7.97	*U* = 14, *P* = 0.83
		Median	33.03	33.63	
		Range	30.08–34.27	28.83–49.13	
	24 dpi	AM ± SD	31.65 ± 11.57	22.96 ± 3.10	*U* = 22, *P* = 0.05
		Median	28.23	22.73	
		Range	21.76–51.73	19.78–26.09	
CD4+ %	8 dpi	AM ± SD	77.37 ± 1.17	70.74 ± 1.67	*U* = 25, *P* = 0.01
		Median	77.24	70.59	
		Range	74.98–79.46	68.14–73.33	
	16 dpi	AM ± SD	74.91 ± 1.56	77.40 ± 1.43	*U* = 22, *P* = 0.06
		Median	75.60	77.36	
		Range	72.70–76.26	75.27–78.73	
	24 dpi	AM ± SD	72.14 ± 6.06	76.55 ± 5.98	*U* = 21, *P* = 0.14
		Median	74.18	78.70	
		Range	62.53–78.17	66.38–81.85	
CD8+ %	8 dpi	AM ± SD	17.74 ± 1.39	21.80 ± 0.57	*U* = 25, *P* = 0.01
		Median	17.14	21.58	
		Range	16.14–19.47	21.16–22.62	
	16 dpi	AM ± SD	14.31 ± 8.43	16.86 ± 2.67	*U* = 13, *P* = 0.99
		Median	16.72	16.40	
		Range	0.15–22.62	13.85–20.15	
	24 dpi	AM ± SD	14.98 ± 2.60	17.89 ± 1.19	*U* = 21, *P* = 0.09
		Median	14.64	17.37	
		Range	12.23–18.69	16.93–19.75	
CD4-/CD8- %	8 dpi	AM ± SD	3.70 ± 0.87	5.69 ± 1.60	*U* = 22, *P* = 0.05
		Median	3.67	6.10	
		Range	2.62–4.90	3.29–7.18	
	16 dpi	AM ± SD	9.27 ± 8.06	4.10 ± 1.22	*U* = 21, *P* = 0.09
		Median	6.67	4.48	
		Range	4.02–23.5	2.45–5.54	
	24 dpi	AM ± SD	7.22 ± 3.61	5.97 ± 2.54	*U* = 17, *P* = 0.41
		Median	6.20	4.93	
		Range	4.13–13.45	3.81–10.3	
CD4+/CD8+ %	8 dpi	AM ± SD	0.43 ± 0.06	0.43 ± 0.09	*U* = 14, *P* = 0.83
		Median	0.41	0.38	
		Range	0.36–0.52	0.38–0.61	
	16 dpi	AM ± SD	0.52 ± 0.29	0.57 ± 0.13	*U* = 12, *P* = 0.99
		Median	0.65	0.58	
		Range	0.01–0.70	0.38–0.72	
	24 dpi	AM ± SD	0.34 ± 0.09	0.48 ± 0.15	*U* = 19, *P* = 0.21
		Median	0.38	0.44	
		Range	0.21–0.42	0.29–0.67	

*Abbreviations*: Group A, immunocompetent infected group; Group C, immunocompetent control group; Group AS immunosuppressed infected group; Group CS, immunosuppressed non-infected group; AM, arithmetic mean; SD, standard deviation

### Histology of the spleen

**Fig. 2:**
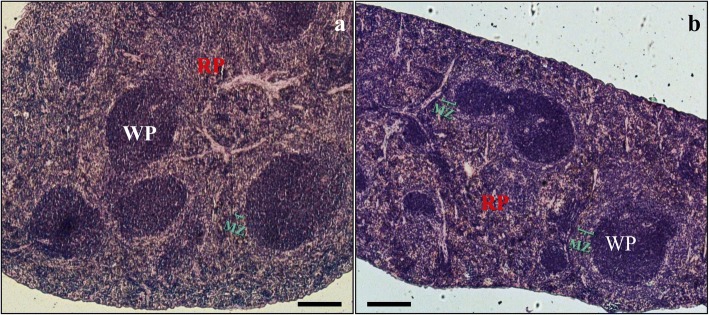
Morphology of control immunocompetent (**a**) and immunosuppressed (**b**) mice spleen. Hematoxylin-eosin staining. *Abbreviations*: WP, white pulp; RP, red pulp; MZ, marginal zone. Magnification of ×50. *Scale-bars*: 200 μm

**Fig. 3:**
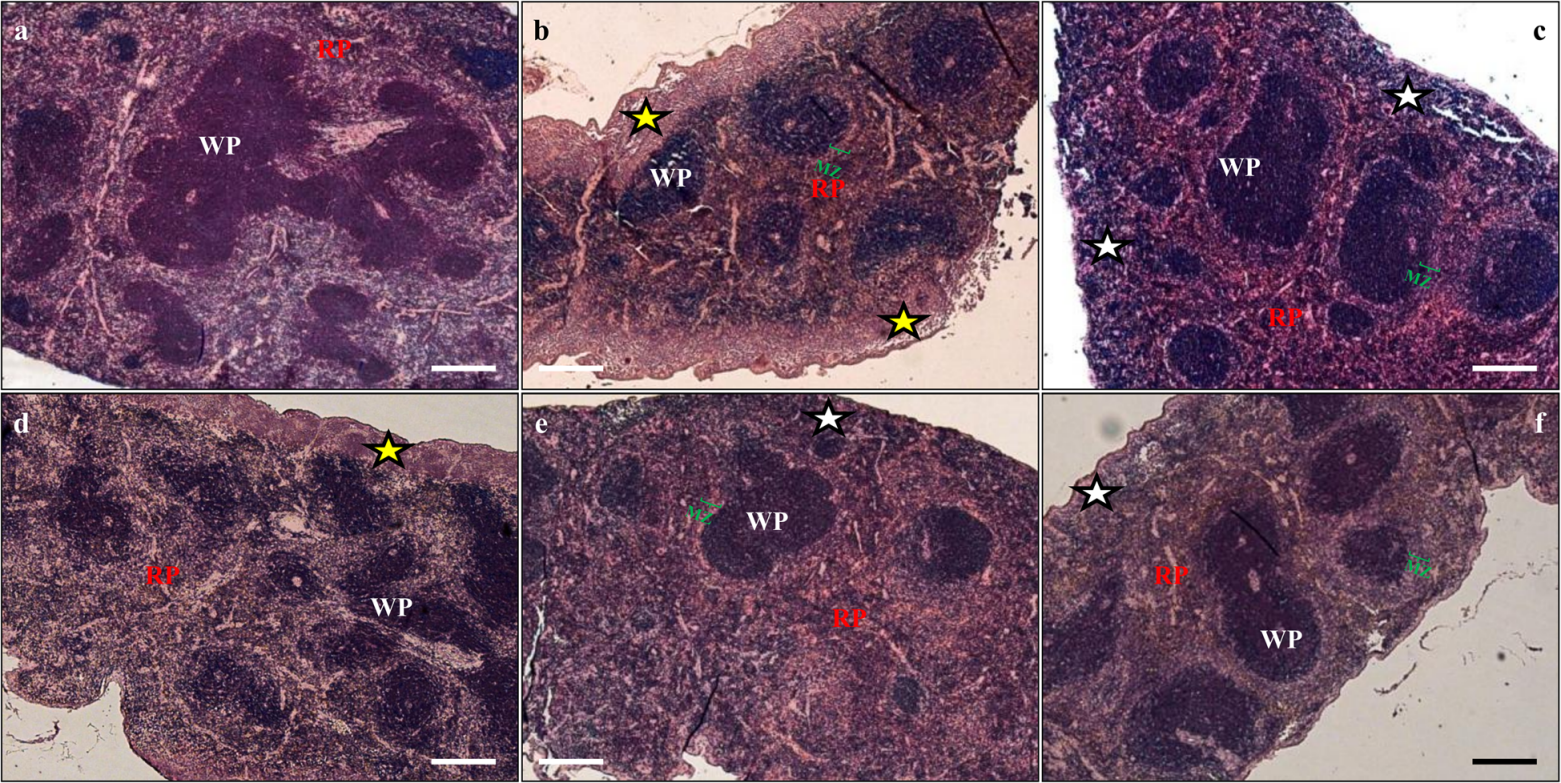
Representative microphotographs showing spleen histological morphology of mice infected by *Acanthamoeba* spp*.* from immunocompetent (**a**-**c**) and (**d**-**f**) with immunosuppressant treatment after 8 (**a**, **d**), 16 (**b**, **e**) and 24 (**c**, **f**) days post-infection. Hematoxylin-eosin staining. *Abbreviations*: WP, white pulp; RP, red pulp; MZ, marginal zone. Yellow and white asterisks indicate subcapsular loose or lymphoid infiltrated area. Magnification of ×50. *Scale-bars*: 200 μm

Figure 2a shows a typical representative histological image of a control mice spleen, with white pulp (WP) consisting of lymphatic nodules with a central artery envelope by PALS, and red pulp (RP) containing venous sinuses and splenic cords. Histology of the spleen after immunosuppressive treatment (Fig. 2b) did not change significantly, although the marginal zone (MZ) was more evident and thicker than in control animals (Fig. 2a). The number and size of lymphatic nodules and structure of the red pulp were very close to that observed in the control mice (Fig. 2a, b). The organs from groups C and CS were compact and enveloped by a thin connective tissue capsule that sent very delicate septa into the spleen parenchyma. After *Acanthamoeba* spp*.* infection (Fig. 3a-f), the observed splenomegaly corresponded with looseness and fragility of the splenic parenchyma. At the beginning of the second week of infection (8 dpi), both in suppressant-treated and control mice (Fig. 3a, d) hyperplasia of the white pulp was observed, and the follicular structure was distorted. In both groups of infected animals (A, AS) the subcapsular area was loose or highly lymphoid infiltrated (yellow or white asterisks, respectively); in the red pulp of these mice, many lymphocytes also occurred. The histological image of the spleen and morphometric measurements showed a decrease in WP/RP ratio at 16 dpi and an increase at 24 dpi in *Acanthamoeba* spp*.* infection (Fig. 3c, e and d, f, respectively). In the amoeba infected and immunosuppressed mice the marginal zone was also more evident (Fig. 3b, c, e, f) than in the control mice (Fig. 2a), which could suggest an increase in the number of unique subset of non-circulating IgM+/IgD− B-cells of the marginal zone, opposite to IgM+/IgD+ follicular B-cells [20].

## Discussion

The course, pathogenesis and clinical picture of a free-living *Acanthamoeba* infection is unclear in both immunocompetent and immunosuppressed hosts. In this experimental model, we assessed the impact of the clinical strain AM 22 of *Acanthamoeba* spp*.* isolated from a patient with low immunity and with atypical pneumonia symptoms, on changes in the immune system in mice depending on the host’s immune status. Immunosuppression at both humoral or cellular levels had different consequences for the host depending on the magnitude and may alter the range of parasites to which the host is susceptible [4].

We noticed a reduction in total spleen mass in *Acanthamoeba* spp.-infected groups (with normal and low immunity) compared to control mice. When analysing acanthamoebiasis during the period of infection, the spleen mass was greater in immunocompetent *Acanthamoeba* spp*.* infected mice at 24 dpi and the weight of this organ decreased at 16 dpi in immunosuppressed infected mice. Likewise, Machado et al. [21], in a *Leishmania chagasi* experimental model, observed a decrease in spleen weight in steroid-immunosuppressed mice. The change in mass of organs located in the peritoneal cavity is one of the indicators of the intensity of peritonitis and may also result from increased cell proliferation [22]. In protozoan parasites, e.g. *Plasmodium* spp., *Trypanosoma* spp., *Leishmania* spp., and *Cryptosporidium parvum* infections, splenomegaly is usually observed, and splenic tissue is a good indicator of immunological activity and parasitic infection in mammals [23]. Gornik & Kuźna-Grygiel [24], in experimental studies on the infection of *Acanthamoeba* spp*.*, observed that the spleen of the infected mice did not differ from the control in the macroscopic view, which coincides with our results. Moreover, in *Acanthamoeba* spp*.* infected spleens, hyperplasia of the white pulp around the arteriolae was found [24].

Knowledge of haematological parameters in mice experimentally infected with *Acanthamoeba* spp. is extremely limited, while in patients with brain, lung, or peritonitis, *Acanthamoeba* spp*.* infections are very diverse. We observed higher lymphocyte and basophile counts in *Acanthamoeba* spp*.* infected immunosuppressed mice than in the control. Moreover, monocyte levels were lower in *Acanthamoeba* spp*.* infected immunocompetent mice than in infected animals with low immunity. Free-living amoeba also induced an increase in platelet count in infected immunocompetent hosts. Data from a previous clinical study showed a reduction in platelets and lymphocytes in a patient with low immunity and *Acanthamoeba* pneumonia [25]. In patients with GAE, there was anaemia, upregulation of leukocytes, stable level of lymphocytes and increased or stable level of platelets [13, 26].

In the early phase of inflammation, phagocytes produce pro-inflammatory cytokines IL-6, IL-8, IL-17 and IFN-γ that can initiate, exacerbate, and maintain the inflammatory process [27]. Antagonistic groups are anti-inflammatory cytokines, including IL-4, produced by Th2 cells that may inhibit the inflammatory process. The roles of IL-4, IL-10, IL-17 and IFN-γ, in *Acanthamoeba* spp*.* pathogenesis are poorly documented. Interleukin-4 is involved in allergic and autoimmune processes [28]. IL-10 acts as an inhibitor of the immune response, blocking the production of Th1-stimulated lymphocytes and stopping the production of inflammatory cytokines, such as IFN-γ, as well as inhibiting IL-4 production by Th2 cells [29, 30]. Many studies on pathogenic protozoa indicate that intracellular *Leishmania* spp., *Plasmodium* spp., and *Trypanosoma cruzi* trigger IL-10, but mechanisms involved in this immune event are still unclear [31–33]. Mattana et al. [34] observed that *Acanthamoeba* trophozoites could induce the production of IL-10 by human mononuclear cells *in vitro*. In this study, we noticed that IL-4 and IL-10 was significantly downregulated in the *Acanthamoeba* spp*.* infected immunosuppressed mice *vs* immunocompetent infected mice at 16 dpi following higher doses of ConA. The host’s immune system may have induced secretion of IL-10 in this study as a response to the infection by *Acanthamoeba* spp*.* Moreover, we found relatively low levels of IL-4 after administration of different doses of ConA except at 16 dpi and in immunocompetent infected mice. In *Acanthamoeba* keratitis, Suryawanshi et al. [35] found an increased production of IL-4, IFN-γ and IL-17A and suggested induction of Th1, Th2, and Th17 cell response in the cornea infection.

Interleukin-17A plays a key role in anti-microbial and anti-fungal defence by inducing cytokines, chemokines that affect neutrophil activation [36, 37]. Some studies have demonstrated that IL-17 cells mediate host defences against Chagas disease, mucocutaneous leishmaniasis, alveolar disease and opportunistic infections such as pneumocystosis, toxoplasmosis and cryptosporidiosis [38–42]. IL-17 is also involved in the pathogenesis of autoimmune diseases and transplant rejection reactions of organs [37]. Suryawanshi et al. [35] suggested that IL-17A production after *Acanthamoeba* spp*.* infection plays an important role in host protection against invading parasites in the human cornea (during *Acanthamoeba* keratitis), although it is not known how it acts as a mediator of systemic inflammatory response, e.g. in amoebic brain and lung infections, and it is not known whether this cytokine participates as a defense mechanism or in the pathogenesis of these diseases. In connection with the multidirectional action of IL-17, it may play a superior role in the cytokine network that regulates immune and inflammatory reactions [39]. In this study, we observed significant expression of IL-17A in *Acanthamoeba* spp*.* infected immunocompetent mice compared to control at 16 dpi and there was significant upregulation of this proinflammatory interleukin in the infected mice with low immunity compared to infected immunocompetent hosts after administration of higher doses of ConA. Moreover, the level of IL-17A significantly differed in infected immunocompetent animals *vs* control mice at 24 dpi.

IFN-γ is a pro-inflammatory cytokine secreted primarily by T and natural killer (NK) cells, playing the role of an inducer of adaptive immune responses against protozoan infections [43]. We noticed that IFN-γ was blocked at 8 dpi and 16 dpi in all study groups regardless of the immune status of the host. On day 24 post-infection, we observed that the *Acanthamoeba* spp*.* infected immunosuppressed mice produced significantly more IFN-γ than infected immunocompetent hosts, as in previous studies on the invasion of *Blastocystis* spp. [44].

Type 1 and 2 cytokines are cross-regulatory groups [45]. Type 1 cytokines (including IFN-γ) favour the development of a strong cellular immune response during parasitic infection whereas type 2 cytokines favour a strong humoral immune response, so IL-4 and IL-10 may decrease the concentration of type 1 cytokines including IFN-γ, which was observed in *Acanthamoeba* spp*.* infection in hosts with normal and low immunity.

Animal models have demonstrated that T cell response seemed to be crucial for parasite control [46]. Flow cytometry analysis from the spleen of *Acanthamoeba* spp*.* infected immunosuppressed mice showed the upregulation of CD3+/CD4+ T cells [%] from the level of the control at 24 dpi. Moreover, in the AS group, we found a significant increase in CD3+/CD8+ T cells (%) at 16 dpi. CD3+CD4-CD8- double-negative (DN) T cells play a key role in regulating the immune responses in transplant rejection, function in graft-*versus*-host disease, autoimmune diseases and also infectious diseases including parasitic infection [47, 48]. DN T cells have a regulatory function similar to regulatory T cells (T_regs_) and secrete CD4-like cytokines including IL-4, IL-17, IFN-γ to exert their T helper function [49]. Liang et al. [50] in HIV positive patients observed that the decrease in DN T cells was correlated with a rapid disease progression. Data from our study shows downregulation of CD3+/CD4-/CD8- T cells (%) in *Acanthamoeba* spp. infected immunosuppressed mice compared to the control at the beginning of infection. CD3+/CD4+/CD8+ double-positive (DP) T cells have been reported in healthy subjects as well as in patients with autoimmune and chronic infectious diseases [51]. Not much is known about their participation in parasitic invasions but in the *Acanthamoeba* spp*.* infected host with low immunity there was an upward trend in the number of DP T cells during the invasion and also a significantly higher DP T cell count in the AS group compared to 24 dpi.

CD3+, CD4+, CD8+ T lymphocytes and CD4+/CD8+ and CD4-/CD8- ratios were also analyzed. In CD3+ T cells, a significant reduction was observed in infected *Acanthamoeba* spp*.* immunosuppressed mice at 8 dpi and a significant peak was observed at 24 dpi. CD4+ T cells play a key defensive role during the infection of parasites; they fully activate CD8+ T cells by destroying infected cells [52]. They are essential for inflammation responses against *Toxoplasma gondii* infection and also take part in the generation of anti-parasitic immunity and immune surveillance during concomitant immunity [53]. It has been demonstrated that CD8+ T cells are essential for protective immunity against the malaria exoerythrocytic cycle in the liver and may play a superior role in the immune response in cutaneous leishmaniasis [54–56]. In *Acanthamoeba* spp*.* infected mice with low immunity we observed an increased CD4+ T cell count at 8 dpi. The subsequent downward trend of CD4+ T and CD8+ T cells during *Acanthamoeba* spp*.* infection may be related to the establishment of the adaptive response to acute infection. Moreover, Gangneux et al. [57] observed that the percentage of CD4+ and CD8+ T cells in the spleen of infected *Leishmania infantum* mice treated with glucocorticoids did not show statistical differences *vs* the control group. Immunosuppressive therapy may interfere with the circulation of immune cells, promote apoptosis of lymphoid cells, and induce downregulation of T cells [21]. Moreover, in *Acanthamoeba* spp*.* infected immunosuppressed hosts, we observed a statistically significant downregulation and an insignificant upregulation of CD4-/CD8- ratios compared to control at 8 dpi, 16 dpi, and 24 dpi. *Acanthamoeba* spp*.* had no significant effect on the CD4+/CD8+ ratio in immunosuppressed mice during infection.

## Conclusions

We have demonstrated that analysis of the immune response and pathogenesis mechanisms of clinical isolates of *Acanthamoeba* spp. in an animal model not only has a purely cognitive significance but above all, may help in the development of effective methods of pharmacological therapy. We observed that the *Acanthamoeba* spp*.* strain isolated from a patient with AML and with pneumophilic properties affected the blood picture in immunocompetent and immunosuppressed mice and induced a change in spleen weight during the infection. Moreover, analysis of anti-inflammatory and pro-inflammatory cytokines produced by splenocytes stimulated with concanavalin A demonstrated that *Acanthamoeba* spp*.* induced a selective Th1, Th2 and Th17 response at later stages of the infection in immunocompetent hosts. In the case of hosts with low immunity *Acanthamoeba* spp*.* elicited robust Th1 cell-mediated immunity without the participation of Th17. We observed suppression of CD8+ and CD4+ T lymphocytes and DN T lymphocyte populations at the beginning, and in the case of DP T-cell in the final phase of *Acanthamoeba* spp*.* infection in hosts with low immunity. Also, CD4+ T lymphocytes and CD3+/CD4+ and CD3+/CD8+ lymphocyte counts during each stage of acanthamoebiasis were shown to be upregulated. Immunological and immunophenotypic results require further analysis and determination of a broader panel of cytokines including IL-1, IL-2 and IL-6, as well as immunophenotypic studies in immunocompetent hosts.

## Abbreviations

A: Immunocompetent *Acanthamoeba* spp*.-*infected mice
AIDS: Acquired immune deficiency syndrome
AK: *Acanthamoeba keratitis*
AM 22: Amoebic strain no. 22
AM: Arithmetic mean
AML: Acute myeloid leukaemia
AS: Immunosuppressed *Acanthamoeba* spp*.* infected mice
BASO: Basophils
C: Immunocompetent uninfected control group mice
CA: Cutaneous acanthamoebiasis
CD: Cluster of differentiation
ConA: Concanavalin A
COX: Cyclooxygenase-1
COX-2: Cyclooxygenase-2
CS: Immunosuppressed uninfected control group mice
DN: Double-negative T cells
DP: Double-positive T cells
dpi: Days post-infection
EOS: Eosinophils
GAE: Granulomatous amebic encephalitis
HCT: Hematocrit
HGB: Haemoglobin
HIV: Human immunodeficiency virus
IFN-γ: Interferon gamma
IgD: Immunoglobulin D
IgM: Immunoglobulin M
IL-1: Interleukin 1
IL-10: Interleukin 10
IL-12: Interleukin 12
IL-17A: Interleukin 17A
IL-2: Interleukin 2
IL-4: Interleukin 4
IL-6: Interleukin 6
IL-8: Interleukin 8
K-W test: Kruskal-Wallis test
LYM: Lymphocytes
mAb: Monoclonal antibodies
MONO: Monocytes
MPS: Methylprednisolone sodium succinate
MZ: Marginal zone
NEU: Neutrophils
NK: Natural killer cells
NN: Non-nutrient agar
NS: Difference non-significant
p: Level of significance
PGE_2_: Prostaglandin E_2_
PLT: Platelets
RBC: Red blood cells
RP: Red pulp
RPMI: Medium Roswell Park Memorial Institute medium
SD: Standard deviation
Th1: Type 1 T helper
Th17: Type 17 T helper
Th2: Type 2 T helper
TNF: Tumor necrosis factor
Tregs: Regulatory T cells
TXB_2_: Thromboxane B_2_
WBC: White blood cells
WP: White pulp
